# To Prevent Plaster from Adhering to Rubber Plates in Vulcanizing

**Published:** 1882-02

**Authors:** 


### TO PREVENT PLASTER FROM ADHERING TO RUBBER PLATES IN VULCANIZING.

Liquid silex, carefully applied with a small *bristle* brush, and allowed to dry before packing the flask, leaves the plate with a durable polish less liable to absorb the fluids of the mouth than is the ordinary finish, especially the palatine surface of plates with deep undercuts. Of course, a first requisite is smooth plaster casts.

Keep the liquid silex in a short bottle with a rubber stopper. Wash the brush in hot water after using. Don't leave the brush in the bottle.

